# Socio-economic factors related to moral reasoning in childhood and adolescence: the missing link between brain and behavior

**DOI:** 10.3389/fnhum.2012.00262

**Published:** 2012-09-24

**Authors:** Simona C. S. Caravita, Simona Giardino, Leonardo Lenzi, Mariaelena Salvaterra, Alessandro Antonietti

**Affiliations:** ^1^Department of Psychology, Catholic University of the Sacred HeartMilano, MI, Italy; ^2^Research Unit in Neuroethics, IRCCS “Eugenio Medea”Bosisio Parini, Italy

**Keywords:** moral reasoning, socio-economic factors, neuroscience, psychological research, moral domain theory

## Abstract

Neuroscientific and psychological research on moral development has until now developed independently, referring to distinct theoretical models, contents, and methods. In particular, the influence of socio-economic and cultural factors on morality has been broadly investigated by psychologists but as yet has not been investigated by neuroscientists. The value of bridging these two areas both theoretically and methodologically has, however, been suggested. This study aims at providing a first connection between neuroscientific and psychological literature on morality by investigating whether socio-economic dimensions, i.e., living socio-geographic/economic area, immigrant status and socio-economic status (SES), affect moral reasoning as operationalized in moral domain theory (a seminal approach in psychological studies on morality) and in Greene et al. (2001) perspective (one of the main approaches in neuroethics research). Participants were 81 primary school (M = 8.98 years; SD = 0.39), 72 middle school (M = 12.14 years; SD = 0.61), and 73 high school (M = 15.10 years; SD = 0.38) students from rural and urban areas. Participants' immigrant status (native vs. immigrant) and family SES level were recorded. Moral reasoning was assessed by means of a series of personal and impersonal dilemmas based on Greene et al. (2001) neuroimaging experiment and a series of moral and socio-conventional rule dilemmas based on the moral domain theory. Living socio-geographic/economic area, immigrant status and SES mainly affected evaluations of moral and, to a higher extent, socio-conventional dilemmas, but had no impact on judgment of personal and impersonal dilemmas. Results are mainly discussed from the angle of possible theoretical links and suggestions emerging for studies on moral reasoning in the frameworks of neuroscience and psychology.

## Introduction

Since the 1990s research on neuroscience has devoted increasing attention to brain processes involved in moral judgments and behaviors by investigating the biological foundations of moral reasoning. In such studies, moral dilemmas, visual sentences, and pictures were used as prompts of moral reasoning and emotions during the scanning of brain activity by functional Magnetic Resonance Imaging (fMRI) and in experiments using non-invasive brain stimulation techniques (e.g., Greene et al., 2001; Koenigs et al., 2009). These methods allowed investigators to identify the brain structures—in particular, the prefrontal and cingulated cortex—which play a role in moral thinking and behavior [for a recent review, see Fumagalli and Priori (2012)]. Starting with the seminal approach devised by Greene et al. (2001), a distinction between two possible brain systems supporting morality has been recurrently proposed. In Greene et al. (2001) experiment, one of the first attempts to identify the neural counterparts of moral judgment that is often quoted in the literature about neuroethics, a series of paired personal and impersonal moral dilemmas were used. The most famous situation described in such dilemmas is the trolley problem, in which the distinction between impersonal and personal dilemmas emerges clearly. The *impersonal* version of the trolley problem (switch dilemma) describes a runaway trolley which is heading for five people who will be killed if it proceeds on its present course. The only way to save them is to hit a switch that will divert the trolley to an alternative track so it will kill one person instead of five. Most people agree that it is right to divert the trolley in order to save five people at the expense of one. In the *personal* version (footbridge dilemma) a trolley threatens to kill five people. You are standing next to a large stranger on a footbridge that spans the tracks, in between the oncoming trolley and the five people. The only way to save the five people is to push this stranger off the bridge onto the track below. He will die if you do this, but his large body will stop the trolley from reaching the others. Most people claim that in this situation engineering the death of that man in order to save the five workers is immoral. In both situations the choice is between five people being killed or one person being killed. Why is the moral choice different in the two scenarios? According to Greene et al. (2001, p. 2106) “the crucial difference between the switch dilemma and the footbridge dilemma lies in the latter's tendency to engage people's emotions in a way that the former does not.” This conclusion was supported by recording participants' brain activity scanned through fMRI. The basic finding was a correlation between the personal vs. impersonal features of the dilemmas and patterns of neural activity in emotion-related brain areas: personal moral dilemmas tended to activate emotion-related cerebral areas to a higher extent than impersonal dilemmas did; by contrast, the activation of brain areas associated with cognitive functioning was higher in impersonal than personal dilemmas.

Building on Greene et al. (2001) paper, the involvement of emotion in moral judgments has been stressed by successive investigations which identified specific neural correlates of reactions and decisions concerning moral issues which are emotionally charged (e.g., Moll et al., 2002; Harenski et al., 2008; Fumagalli et al., 2010). This supported the notion that in moral judgment and behavior two brain networks are involved, each associated with a distinctive attitude or form of processing: cognitive vs. emotional, reasoning-based vs. intuition-based, and explicit vs. implicit (Haidt and Joseph, 2004; Huebner et al., 2008; Moll and Schulkin, 2009; Bennis et al., 2010).

Identification of specific neuronal structures that contribute to moral reasoning and behavior strengthened the hypothesis of some innate neurobiological roots of morality (e.g., Haidt, 2001; Hauser, 2006), that could even explain the possible universal facets of moral reasoning. In this vein, in psychological research on morality and its development one of the most prominent theories is the moral domain theory (e.g., Turiel, 1983). According to this approach, moral knowledge is built and organized in separate domains which are related to different kinds of rules: *moral rules*, which are aimed at preserving the other's physical, psychological and social well-being (e.g., the rule forbidding kicking or hitting a classmate), and *socio-conventional rules*, which are set by authorities to better coordinate social interpersonal relationships in the social systems in which they are located (e.g., the school rule on calling the teachers by their first name). In reason of their own nature, moral norms are perceived by people as worthy by themselves and universally valid, independently of authorities' dictates and capacity to punish transgressors. On the other hand, by reason of their social purpose, socio-conventional rules are usually conceived to be closely dependent on authorities' capacity to guarantee respect of the norms and to be valid only in societies and organizations where authorities establish rules. As regards rule transgressions, people usually evaluate as serious and do not accept the breaking of moral rules, because of their harmful consequences for other(s), whereas, they judge breach of socio-conventional rules, which has no consequences of harm or unfairness, as less serious and acceptable under some conditions, for instance when they happen outside the context in which the rule has been formally stated (Turiel, 1983; Smetana, 1988; Smetana and Braeges, 1990; Smetana et al., 1993).

The distinction between moral and socio-conventional transgressions may be grounded on a different activation of emotions, since in moral rule violations the person empathizes with the victim, but empathy is not elicited by socio-conventional transgressions (Churchland, 2011). In the perspective of domain theory, the situations described in the personal and impersonal dilemmas proposed by Greene et al. (2001) can be considered as specific instances of transgressions of the moral rule which forbids killing or harming another person, even if her/his sacrifice could be advantageous for others. Therefore, personal and impersonal dilemmas assess the domain of moral rules by establishing a distinction between moral rule transgressions which elicit strong emotional-empathic reactions and moral rule violations which are less emotionally-empathically connoted.

Several studies have also provided some evidence that children make judgments and classify actions in terms of the moral or Socio-conventional domains from a very young age (e.g., Smetana, 1984; Tisak and Turiel, 1988; Smetana and Braeges, 1990; Smetana et al., 1993; Zelazo et al., 1996; Caravita et al., 2009; Gasser and Keller, 2009). For instance, from the age of 3 years children are aware of the consequences of moral actions and they evaluate situations such as stealing or hitting without provocation as wrong because of their intrinsic unfairness (Helwig et al., 2001); 4-year-old children are also able to distinguish between basic moral and Socio-conventional events (Smetana, 1981; Smetana and Braeges, 1990).

The precocity of making distinctions in evaluating the seriousness of rule transgressions of different domains is somehow consistent with the hypothesis of certain innate foundations or precursors of moral reasoning, such as an intuitive sense of moral wrongness (Haidt, 2001). Nevertheless, some studies in the framework of moral domain theory also support the notion that the evaluations of rule transgressions, even moral-rule violations, can be affected by differences in socio-economic conditions and cultural background (Sachdeva et al., 2012).

As regards socio-economic factors, notwithstanding the conception of morality as potentially grounded in biological structures and innate components, some neuroscientific studies have also provided evidence that neuronal structures and networks can be influenced by socio-economic dimensions. Variation in socio-economic status (SES) levels have been found to be associated with variation in brain serotonergic responsivity, and therefore, may also be related to differences in the prevalence of diseases and problematic behaviors, including aggression (Manuck et al., 2005). Gianaros and Manuck (2010) reviewed several studies, finding that indicators of socio-economic position (SEP) are connected with the functionality of monoamine neurotransmitter systems and to the activity and morphology of brain circuitries that are also involved in risk of health conditions. In an fMRI study (Lane et al., 2009), people reporting lower levels of subjective SEP displayed a reduced volume of gray matter in the rostral area of the anterior cingulate cortex. This area is implicated in the regulation of emotions and in organizing both physiological and behavioral reactivity to psycho-social stressors. Poverty status is also associated with individual neuro-cognitive performance in language, executive functions and memory in children and adolescents [e.g., Farah et al., 2004; Noble et al., 2005; for a review, see Farah et al. (2007)], suggesting that lower SES levels are connected with variation in the activity of some areas of the prefrontal cortex. In a research project on reading abilities, Noble et al. (2006) found that SES systematically modulated the associations between reading-related brain activities in the perisylvian and left fusiform areas and the phonological language skills of children with equivalent phonological skills. Altogether, this literature supports the hypothesis that socio-economic conditions may affect the function, and perhaps the development, of at least some of the brain areas and structures associated with emotion and cognition.

Moving from neuroscience to psychological researching on moral development, socio-economic factors have been found to affect moral values and moral processes of evaluating situations and taking behavioral decisions [for a recent review, see Sachdeva et al. (2012)]. For instance, the economic background of individuals helps to define the worth that is attributed to values or rules. The process is particularly evident for rules conceivable as socio-conventional. Haidt et al. (1993) carried out a study on the perception of moral violations that included (1) transgressions of rules preserving the other's well-being (i.e., moral rules, according to the moral domain framework) and (2) disrespect situations that were not harmful to others but involved the infringement of socio-conventional rules, such as using the national flag to clean the toilet. People of low SES evaluated the latter kind of situations as morally wrong more than did high-SES people, who were more likely to consider those behaviors as socially inappropriate and not as moral violations. In accordance with these findings, values and evaluations of what is moral seem to vary according to the differences in the social position of individuals, even those belonging to the same cultural context or country: for instance, the Indian social system of castes that are characterized by different standards in the conceptualization of what is morally right or wrong [for a review, see Sachdeva et al. (2012)].

Besides socio-economic factors, even cultural aspects can modulate moral reasoning. For instance, the identity of the person who is damaged by a behavior and of the agent of that behavior can define the moral acceptability of the action in some cultures but not in others. In fact, in some populations of New Guinea harming or killing a member of the same clan may be not acceptable, but if the victim belongs to a rival clan it becomes a moral obligation (Read, 1955). Among studies using the trolley problem paradigm or similar scenarios, Uhlmann et al. (2009) found that the evaluation of the sacrifice of the innocent victim as acceptable varied according to the identity of the victim (e.g., Iraqis vs. American civilians). In the same vein, Sachdeva et al. (2012) reported findings from a still unpublished study showing that Indian university students accepted the harmful action in the footbridge problem more readily when the agent was described as a member of the warrior caste than as a component of the priestly or scholarly caste. In the light of their own research data, Sachdeva et al. (2012) also suggested that, for components of cultures that are group- or duty-based, the distinction between the personal and impersonal versions of the trolley dilemma may be not as salient as for people belonging to individual- or rights-based cultural systems.

Cultural differences are mirrored in immigrant populations. Studies on morality and immigrant families have found some evidence that in child-rearing practices immigrants give emphasis to adopting the values of the host culture while maintaining the values of their own cultural heritage (Liu, 2009). Accordingly, immigrant parents modify children's school-related literacy activities to reflect their existing cultural beliefs and practices, including moral teaching (Perry et al., 2008). Therefore, in building their social identity immigrants refer to models from the host society but also attribute relevance to their cultural origins for moral dimensions.

In a tighter perspective, even living in urban or rural areas, that is, different socio-geographic/economic areas with related differences in density of the population, in economic activities, in socio-economic opportunities and infra-structures (e.g., OECD, 1994; EC, 2005; UNECE, FAO, OECD, and World Bank, 2005; Marcellini et al., 2007), has been found to have some influence on moral values and reasoning (e.g., Yagnik and Teraiya, 1999). For instance, in a study on early adolescents, rural youngsters were found to be higher in moral reasoning than peers from urban areas (Sahoo, 1985). Another research project on 10–28-year-olds, however, showed that people from villages tended to justify their moral decisions mainly in the norm-following and utilitarian modes, whereas, older urban youngsters showed a tendency to use deontological and perfectionistic justifications (Nisan and Kohlberg, 1982).

Given this background, investigating the impact of socio-economic, cultural and rural vs. urban characteristics of the living environment on morality appears to be a promising and important topic for neuroscientific research on moral reasoning. To our knowledge, however, no studies on neurobiology of morality have also included the investigation of differences in living socio-geographic/economic area, cultural, and economic conditions until now. Furthermore, most neuroscientific studies on morality involved adult healthy subjects and patients, whereas, only a very few studies have been realized on children and adolescents. (e.g., Eslinger et al., 2009) and we could not find any study that also considered possible differences between children/adolescents and adults in the brain counterparts of moral reasoning. The investigation of morality among children and adolescents represents, however, an important line of research in the psychology area (Kohlberg, 1981; Killen and Smetana, 2006). This framework highlights the necessity of examining features of moral reasoning and emotions in childhood and adolescence, not assuming the characteristics of morality in adulthood as a universal model of how morality works in children and adolescents.

Recently, overcoming the gap between neuroscientific studies and psychological studies on morality in childhood and adolescence has been proposed as a relevant challenge for future research (Killen and Smetana, 2008). Filling such a gap requires consideration of the differences in both theoretical background and content, as well as in methods. Consequently, we aimed to make a preliminary contribution towards bridging the gap between research on neurobiological foundations on morality and psychological research on morality by investigating whether and how certain socio-economic and cultural factors affect the moral reasoning of children and adolescents, as conceptualized and assessed in classical neuroscientific studies and in the moral domain theory. Furthermore, we aimed to test new tasks (Antonietti et al., 2012) which can be employed to assess moral reasoning because of their particular characteristics, in both neuroscientific and psychological research, so helping to construct a bridge between the two research fields.

In consideration of such purposes, we devised a research project aimed at investigating the influence of socio-geographic/economic area, cultural, and socio-economic factors on children's and adolescents' moral reasoning by assuming the theoretical background of the moral domain theory and the personal vs. impersonal distinction proposed by Greene et al. (2001) as conceptual frameworks.

First, we believe that personal and impersonal dilemmas used in neuroscientific research represent two kinds of transgression of moral rules: transgressions which imply direct contact with the victim vs. transgressions which imply indirect contact with the sacrificed person and that can be assumed to be less able to activate emotions. We expected that the transgression of moral rules would be judged as less acceptable than the infringement of socio-conventional rules and that the harmful actions described in personal dilemmas would be evaluated as more serious and less acceptable than the actions described in impersonal dilemmas, since personal dilemmas activate emotions to a higher degree than do impersonal dilemmas.

Second, we aimed at investigating whether certain socio-geographic/economic area related, cultural, and socio-economic factors affect not only the evaluations of acceptability of transgressions of socio-conventional vs. moral rules, but also the judgments of acceptability of the harmful actions as described in personal and impersonal dilemmas similar to those employed by Greene et al. (2001); in the literature, such dilemmas have never been considered in association with socio-economic and cultural dimensions. As far as moral and socio-conventional dilemmas are concerned, previous literature (see Sachdeva et al., 2012) showed that socio-economic factors are influential on evaluations of acceptability of transgressions of both moral and socio-conventional rules, but they are especially associated with variation in the evaluation of socio-conventional norm violations. Therefore, we expected that some differences would emerge in the evaluations of acceptability or rule transgressions because of differences in the socio-geographic/economic area in which participants lived, their immigrant status and their SES, and we conjectured that these dimensions were more influential for evaluations of socio-conventional rule infringements than for those of moral rule transgressions. As far as personal and impersonal dilemmas are concerned, because this is one of the first studies exploring how the evaluations of these kinds of situations can be affected by living socio-geographic/economic area, cultural, and socio-economic differences and the first one carried out on children and adolescents and in the Italian context, we were more speculative in terms of formulating our hypotheses. We mainly conjectured that the investigated factors were likely to be somehow influential on moral judgment, and in particular on impersonal dilemmas, that are less emotionally activating.

## Materials and methods

### Participants

Participants (Table 1) were 81 fourth-graders (M = 8.98 years; SD = 0.39), 72 seventh-graders (M = 12.14 years; SD = 0.61), and 73 tenth-graders (M = 15.10 years; SD = 0.38). In the Italian school system grade 4 is one of the two last grades of primary school, grade 7 is in middle school, and grade 10 is in high school. The three groups, equivalent for numbers of participants, were chosen so as to represent three different school levels (primary, middle, and high) of the Italian system. The same interval of 2 years separated the groups and the selected school grades prevented us interviewing students who were attending the first year of a school level (students attending the first year might not yet be accustomed to the new school level they were attending) but on the other hand we were prevented from recruiting students who were attending the last grade of the school level in which they were enrolled (students attending the last year are usually involved in preparation for formal or informal examinations which are often required in the last grade, and this, besides making teachers less available to take part in the research project, could interfere with the execution of the research tasks, for instance allowing researchers to test students at less favorable times in the school day).

**Table 1:** **Sample characteristics as percentages**.

	**Primary school (*n* = 81) Mean age: 8.98 years (SD 0.39)**	**Middle school (*n* = 72) Mean age: 12.14 years (SD 0.61)**	**High school (*n* = 73) Mean age: 15.10 years (SD 0.38)**	**Total (*N* = 226)**
**GENDER**				
Boys	59.3	55.6	52.1	55.8
Girls	40.7	44.4	47.9	44.2
**AREA**				
Urban	46.9	48.6	52.1	49.1
Rural	53.1	51.4	47.9	50.9
**IMMIGRANT STATUS**				
Native	79.0	76.4	90.4	81.9
Immigrant	21.0	23.6	9.6	18.1
SES^*^				
Middle-low	27.2	27.8	6.8	20.8
Middle	54.3	65.3	48.0	55.8
Middle-high	16.1	6.9	45.2	22.5

Note:

* Two missing data in primary school.

Participants attended two primary schools, two middle schools, and two high schools in northern Italy. For each of the three school levels, one school was situated in the city of Milan and the other one in a small village or town in the province of Brescia. After Rome, Milan is the second largest city in Italy, with more than 1,324,000 inhabitants and a further 3,000,000 people who live in the metropolitan surrounds. The urban area of Milan is mainly industrial and is the most important industrial center in Italy. At the end of 2011, immigrants resident in the city of Milan numbered more than 217,300, i.e., 16.4% of all residents in Milan (Statistics of the City Hall of Milan, 2010). After Milan, in Lombardy the second biggest city is Brescia with around 193,800 inhabitants. The province of Brescia is very large and includes many small towns and villages, which are mainly characterized by rural and farm activities. Schools that were recruited in the area of Brescia were situated in a rural village (Borgo San Giacomo) and in a small rural town (Manerbio) in the province. Inhabitants of Borgo San Giacomo number about 5500, 17.6% of whom are immigrants. People resident in Manerbio number about 13,270, with around 1850 (14% of the population) immigrants living in the town.

In the participant sample, immigrants numbered 18.1%: they constituted 21% of the primary school pupils, 23.6% of the middle school students and 9.6% of the high school participants. Among immigrant participants, Asian children totaled 55.8%: 23.3% of immigrant students came from Africa, 11.6 % from other European countries, and 9.6% were from South America. When we considered the urban area of Milan and the rural area of Brescia separately, immigrant participants constituted 17.4% of students from the Milan area and 18.4% of the subsample from the Brescia area.

With regard to SES status (two missing data), 20.8% of participants' families were of low-middle SES, 55.8% of middle SES, and 22.5% of middle-high SES.

### Procedure

Before we allocated dilemmas (see next section), personal and impersonal dilemmas were mixed and then divided into two sequences so that the personal and impersonal versions of the same dilemma were not included in the same sequence. Even the moral and socio-conventional dilemmas were mixed and then organized in two sequences. In this way we obtained four sequences of dilemmas: sequences A, B of moral and socio-conventional dilemmas and sequences C, D of personal and impersonal dilemmas. The four sequences were then alternated and their order was reversed in order to obtain two administration protocols, in which sequences were ordered as follows: protocol 1 = sequences A, C, B, and D; protocol 2 = sequences D, B, C, and A. This strategy allowed us to start protocol 1 with one of the two sequences of moral and socio-conventional dilemmas, whereas, in protocol 2 the starting sequence was one of the two personal and impersonal dilemma sequences. Furthermore, the sequence of moral and socio-conventional dilemmas that was first presented in protocol 1 was presented second in protocol 2; the same happened for the two personal and impersonal dilemma sequences.

Each protocol was administered to half of the classes on each school level and area (i.e., urban vs. rural areas). Protocols were group-administered in the participants' classrooms during the school day in a single session of around 90 min per classroom. A research assistant supervised the protocol administration, explaining the goals of the study and how to fill in the dilemma protocol; she answered any question that was raised by participants. In primary school classes the research assistant also read aloud each of the dilemmas to give children the time to answer the dilemma question. A week after the protocol administration a make-up session was carried out to administer the protocol to students who were absent on the administration day. The study was authorized by the ethical committee of the IRCCS (Scientific Institute for Research, Hospitalization, and Health Care) “Eugenio Medea.” Head-teachers and principals of schools and class committees of teachers allowed the participation of schools and classes in the study. Parents (or legal guardians) of children participating in the study were informed of the aims and procedure of the research project by means of a letter that was sent by schools and delivered in the classrooms by the teachers. In the letter parents were also informed that participation in the study was not mandatory and they were invited both to sign the consent form that was attached to the letter and to give back the signed form to the class teachers within 2 weeks if they consented to their children's participation in the study. In the letter the possibility to contact the researcher (whose mail and phone contact address were provided) in order to obtain more information was mentioned. All children of the involved classes obtained active consent for their participation in the study. At the beginning of the session, however, participants were also informed that their participation in the study was not obligatory, that it was not a curricular activity but an activity for purposes of research only, that they had the right to decide not to participate and that they could withdraw from the study at any time without any consequences. Then, before starting to fill in the measures they were requested to give oral consent to participation in the study and they were allowed to freely interrupt the administration at any time. Only five students (2.16% of pupils in the classes) decided not to participate in the study.

### Instruments

#### Personal vs. impersonal moral dilemmas

A series of eight pairs of dilemmas to be administered to primary school children and a corresponding series of dilemmas to be administered to older students were used (Antonietti et al., 2012). In both series the distinction between the personal and the impersonal versions is based on a unique, unequivocal criterion and possible confounding variables are discarded (Antonietti, 2011). Each dilemma has two parallel versions, so that a direct match between the personal and impersonal scenarios of each dilemma is possible, the contextual information being the same for each dilemma, apart from the aspect which specifically distinguishes between personal and impersonal versions. All dilemmas share the fact that the “helper,” in order to produce a benefit to another child, has to operate so that harm, loss or disadvantage affects a third child/adolescent who is an innocent victim. In impersonal dilemmas the relationship with such a “victim” is indirect, namely, the helper does not interact face-to-face with the victim; in personal dilemmas the helper-victim relationship is direct, that is, the helper touches, looks at and/or speaks to the victim.

The formal features of the dilemmas are as follows: short text; easily understood; not involving knowledge of specific social norms; not describing dramatic and emotionally impressive situations. Furthermore, all dilemmas share the following “narrative” characteristics: They always and only involve children (as far as the moral aspect is concerned); the victim is always unaware (otherwise he/she might decide to sacrifice him/herself) and cannot avoid being involved in the action carried out by the helper; the helper cannot sacrifice her/himself instead of the victim; the action performed by the helper always has a certain outcome; the helper gains no direct advantage or suffers damage as a consequence of her/his action.

In order to insure equivalence and exclude other possible confounding variables, the impersonal and personal versions of each dilemma are similar with respect to the number of words, the number of contrastives (such as “but,” “however,” and “conversely,” which might be influential since they stress the opposition between the two outcomes of the vignette), the degree of difficulty of the wording and of the syntactic structure, the degree of responsibility of the victim and of the beneficiary and the basic script (the helper may damage the victim to produce a benefit to the beneficiary).

The same basic structure of a pair of parallel dilemmas served for the series for primary school students and the corresponding series for older students (in the Italian school system middle and high schools share the same main characteristics). The differences between the versions devised for the two age levels rely on the kind of objects and situations mentioned in the dilemmas, in order to match what usually occurs in, respectively, children and adolescents' school and everyday experiences. In both the series, in half of the dilemmas characters are girls and in half boys. Examples of pairs of dilemmas are as follows:

“Marco is hitting Giorgio very hard. You strike Marco with a football to stop him. Marco starts to cry and Giorgio succeeds in escaping.”

(*Primary school—Personal version*)

“Nicola is hitting Luca very hard. You see that a football about to hit Nicola on the head and you don't warn him. Nicola is struck by the ball and starts to cry and Luca succeeds in escaping.”

(*Primary school—Impersonal version*)

“Anna spreads rumors about Elisa and writes nasty things about Elisa in her own diary. You want Elisa to know what is happening. In front of Elisa you force Anna to give you her diary and read aloud what she has written about Elisa.”

(*Middle and High school—Personal version*)

“Luisa spreads rumors about Caterina and writes nasty things about Caterina in her own diary. You want Caterina to know what is happening. You make Caterina find Luisa's diary and read what Luisa has written about her.”

(*Middle and High school—Impersonal version*)

Each dilemma ends with the question: “Is it right to do so?” The respondent has to check the box corresponding to the response “It is right” (coded as 1) or “It is wrong” (coded as 0) written below the text of the dilemma. Total scores of the sub-series of personal and impersonal dilemmas were the sum of the dilemma answers, with higher scores corresponding to admission of the damage to the innocent victim. In all school-level groups, reliability (Cronbach's alpha) of both the sub-series of personal and impersonal dilemmas was low but still acceptable, given the meaningful content of the measure and the small number of items (Schmitt, 1996). Reliability was better for middle school (personal dilemmas: α = 0.59; impersonal dilemmas: α = 0.64) and high school students (personal dilemmas: α = 0.57; impersonal dilemmas: α = 0.64) than for primary school children (personal dilemmas: α = 0.55; impersonal dilemmas: α = 0.50).

#### Moral vs. socio-conventional dilemmas

A series of 20 moral and a series of 20 socio-conventional dilemmas were created (Antonietti et al., 2012) to assess the perception of rules as breakable. Each item describes a situation in which a child is breaking a school rule that has been explicitly stated. In moral dilemmas the broken school rules are rules aimed at preserving the other's rights and psychological or physical well-being; in socio-conventional dilemmas the broken rules are designed to preserve the social order. Moral and socio-conventional dilemmas all involve only characters of the same age and of the same school-level of respondent. In both series, the child reading the situations assumes the perspective of the main character of the stories. In half of the dilemmas other characters are girls and in half boys. Dilemmas of the two series are equivalent in terms of the word number and grammatical structure. Sample dilemmas are as follows:

“In your school there is a rule that you must not take other children's things. One day at school you are in the cafeteria and you force John to give you his lunch and you eat it in front of him.”

(*Primary school—Moral dilemma*)

“In your school there is a rule that children must stand up when an adult enters the classroom. A morning a school janitress enters into the classroom and you don't stand up because you are aiming to draw.”

(*Primary school—Socio-conventional dilemma*)

“In your school there is the rule forbidding damage to the belongings of others. One day at school during the break time you rip up the assignment of one of your classmates because she has obtained a better mark than you.”

(*Middle and High school—Moral dilemma*)

“In your school there is a rule forbidding you to leave books outside personal lockers at the end of lessons. One morning, you are in a hurry to get out of school and you leave your history book on your desk.”

(*Middle and High school—Socio-conventional dilemma*)

For personal and impersonal dilemmas alike, each moral and socio-conventional dilemma is followed by a question asking whether the rule transgression is right (coded as 1) or wrong (coded as 0).

For both series of dilemmas the total score was obtained by summing the dilemma scores. The reliability of both series was acceptable among primary school children (moral dilemmas: α = 0.67; socio-conventional dilemmas: α = 0.83), good among both middle school (moral dilemmas: α = 0.91; socio-conventional dilemmas: α = 0.92), and high school students (moral dilemmas: α = 0.91; socio-conventional dilemmas: α = 0.90).

#### Immigrant status and SES

At the beginning of the anonymous protocol including the dilemma measures (see Procedure) participants were asked to answer eight demographic questions in order to assess gender, age in years, immigrant status (two questions), and family SES (four open-ended questions).

The two questions assessing participants' immigrant status consisted of a closed-ended question that requested participants to indicate whether they were *Italian* or *foreign*^1^, i.e., non-Italian citizen, and an open-ended question asking for the name of their birth country if they were not Italian (i.e., “If you are foreign, please report your birth country”).

The four open-ended questions assessing families' SES asked participants to report the job and qualifications of their father and mother. When one or both parents were dead or not living with their children, participants were asked to answer with reference to the adult care-givers who had legal responsibility for them. Based on statistical information on the average incomes of jobs provided by the Italian National Institute of Statistics, information on fathers' jobs and mothers' jobs was then classified in three categories of low-middle income, middle income, and middle-high income. Again, the two variables of fathers' and mothers' income levels were cross-referenced with each other and with the information on parental qualifications, in order to classify participants' families in SES categories. Although data on parental qualification were also considered, the allocation of families to SES categories was carried out by examining data on parental jobs. Data on parental qualifications provided supplementary information useful for better evaluation of the SES status of families but were not essential for assessing families' SES. We adopted this procedure because we were assessing the SES of participants, and not the socio-cultural level of their families, and in the literature (e.g., Pineo et al., 1977) SES is assessed with reference to parents' jobs. Following this procedure, we distinguished three SES categories of participants' families: families of low-middle SES level, that is, in which one or both parents did not have a job or had a job with low wages (and had only obtained a primary or middle school, that is, the obligatory school level in the Italian school system, qualification); families of middle SES level, based on at least one of the parents having a middle income level (and qualifications at least at high school level); families of middle-high SES level, whereby one or both the parents had a job with a high income (and in some cases an undergraduate degree; for families in this category, however, parents had high qualifications post-school).

## Results

### Strategy of analysis

Data were analyzed by Repeated Measures Analyses of Variance, with Student-Newman-Keuls post-hoc test. ANOVAs were computed by assuming the total scores in the series of, respectively, personal vs. impersonal and moral vs. socio-conventional dilemmas as dependent variables and the Kind of dilemma as within-subject factor, Gender as between-subject factor and, depending on the case, Area or Immigrant status or SES as third (between-subject) factor. The degrees of freedom vary in the analyses because of some missing data.

### Effects of kind of dilemma and gender

As far as the personal vs. impersonal dilemmas were concerned, in all ANOVAs (Tables 2–4) the effects of the Kind of dilemma were statistically significant, with lower scores in personal than impersonal dilemmas. Also, the effects of Gender were always statistically significant: girls obtained lower scores than boys (Tables 2–4).

**Table 2:** **Mean scores (and standard deviations) of personal vs. impersonal and moral vs. socio-conventional dilemmas in ANOVA including area as fixed factor**.

**Group**	**Personal dilemmas**	**Impersonal dilemmas**	**Moral dilemmas**	**Socio-conventional dilemmas**
Urban area	1.88 (1.47)	2.56 (1.89)	2.23 (3.69)	4.92 (4.97)
Boys	2.24 (1.41)	2.85 (1.87)	2.88 (4.14)	5.46 (5.16)
Girls	1.33 (1.41)	2.12 (1.85)	1.28 (2.67)	4.15 (4.63)
Rural area	1.71 (1.54)	2.38 (1.61)	1.12 (2.52)	3.45 (4.62)
Boys	1.81 (1.53)	2.61 (1.63)	1.35 (2.35)	3.60 (4.72)
Girls	1.60 (1.55)	2.12 (1.55)	0.87 (2.69)	3.30 (4.56)
Total	1.79 (1.50)	2.47 (1.75)	1.68 (3.21)	4.20 (4.86)
Boys	2.03 (1.48)	2.74 (1.76)	2.18 (3.51)	4.61 (5.03)
Girls	1.48 (1.49)	2.12 (1.68)	1.06 (2.67)	3.70 (4.59)

**Table 3:** **Summary of ANOVA for personal vs. impersonal and moral vs. socio-conventional dilemmas, including area as fixed factor**.

	**Personal vs. impersonal dilemmas**			**Moral vs. socio-conventional dilemmas**		
	**df**	**F**	**η^2^**	**df**	**F**	**η^2^**
Kind of dilemmas	1, 207	44.19^***^	0.180	1, 221	123.54^***^	0.360
Area	1, 207	0.26	<0.001	1, 221	5.50^*^	0.024
Gender	1, 207	8.67^**^	0.040	1, 221	3.47^†^	0.015
Kind of dilemmas × area	1, 207	0.03	<0.001	1, 221	0.71	0.003
Kind of dilemmas × gender	1, 207	0.08	<0.001	1, 221	0.29	0.001
Area × gender	1, 207	1.40	0.010	1, 221	1.16	0.005
Kind of dilemmas × area × gender	1, 207	1.26	0.010	1, 221	0.02	0.001

Note:

* *p* < 0.05,

** *p* < 0.01,

*** *p* < 0.001,

† *p* < 0.10.

**Table 4:** **Mean scores (and standard deviations) of personal vs. impersonal dilemmas and moral vs. socio-conventional dilemmas in ANOVA including immigrant status as fixed factor**.

**Group**	**Personal dilemmas**	**Impersonal dilemmas**	**Moral dilemmas**	**Socio-conventional dilemmas**
Native	1.84 (1.53)	2.54 (1.73)	1.55 (3.01)	4.29 (4.97)
Boys	2.08 (1.53)	2.85 (1.72)	2.01 (3.28)	4.61 (5.15)
Girls	1.54 (1.49)	2.15 (1.67)	0.98 (2.55)	3.92 (4.73)
Immigrant	1.57 (1.37)	2.14 (1.84)	2.32 (3.98)	3.77 (4.29)
Boys	1.83 (1.27)	2.26 (1.89)	2.96 (4.40)	4.61 (4.54)
Girls	1.14 (1.46)	1.93 (1.82)	1.47 (3.26)	2.65 (3.77)
Total	1.79 (1.50)	2.47 (1.75)	1.68 (3.21)	4.20 (4.85)
Boys	2.03 (1.49)	2.74 (1.76)	2.18 (3.51)	4.61 (5.03)
Girls	1.48 (1.49)	2.12 (1.68)	1.06 (2.67)	3.70 (4.59)

With regard to moral vs. socio-conventional dilemmas, as reported in Tables 2–4, the main effect of the Kind of dilemma was significant in all the ANOVAs: scores were lower in moral than socio-conventional dilemmas. When we performed separate analyses in the three school-level groups^2^, main effects of Kind of dilemma were similar to the findings obtained in the overall sample. As far as the personal vs. impersonal dilemmas were concerned, in all ANOVAs (not reported here to save space) the effects of the Kind of dilemma were statistically significant (*p*_s_ < 0.05): Scores were lower in personal than in impersonal dilemmas. With regard to moral vs. socio-conventional dilemmas, the main effect of the Kind of dilemma was significant in all three school levels analyzed separately (*p*_s_ < 0.01), with lower mean scores for the moral than for the socio-conventional dilemmas.

The main effect of Gender was marginal in the ANOVA including the Area as fixed factor, but significant in ANOVAs including Immigrant status and SES: girls scored lower than boys.

### Effects of urban vs. rural area

#### Personal vs. impersonal dilemmas

Area (Table 2) did not significantly influence responses and all interaction effects were non-significant (Table 3).

#### Moral vs. socio-conventional dilemmas

Scores differed significantly across the areas, with the lowest mean scores for youngsters living in the rural area in comparison with peers living in the urban area (Table 2). The size of the effect for Area was not very high, however. None of the interaction effects was significant (Table 3).

### Effects of immigrant status

#### Personal vs. impersonal dilemmas

Being Italian or an immigrant (Table 4) failed to affect responses significantly and the native vs. immigrant status did not interact significantly with the other variables (Table 5).

**Table 5:** **Summary of ANOVA for personal vs. impersonal and moral vs. socio-conventional dilemmas, including immigrant status as fixed factor**.

	**Personal vs. impersonal dilemmas**			**Moral vs. socio-conventional dilemmas**		
	**df**	**F**	**η^2^**	**df**	**F**	**η^2^**
Kind of dilemmas	1, 207	22.66^***^	0.100	1, 221	50.22^***^	0.185
Immigrant status	1, 207	1.92	0.010	1, 221	0.01	<0.001
Gender	1, 207	4.52^*^	0.020	1, 221	3.86^*^	0.020
Kind of dilemmas × immigrant status	1, 207	0.09	<0.001	1, 221	5.27^*^	0.023
Kind of dilemmas × gender	1, 207	0.13	0.001	1, 221	0.01	<0.001
Immigrant status × gender	1, 207	0.05	<0.001	1, 221	0.43	0.002
Kind of dilemmas × immigrant status × gender	1, 207	0.86	0.004	1, 221	0.45	0.002

Note:

* *p* < 0.05,

*** *p* < 0.001.

#### Moral vs. socio-conventional dilemmas

The interaction effect of Kind of dilemma × Immigrant status was significant (Table 5). Both Italians' and immigrants' scores were lower in moral than in socio-conventional dilemmas, but immigrants obtained scores concerning moral rules slightly higher than Italians, whereas, Italian students scored higher than non-Italian pupils in socio-conventional dilemmas (Table 4). Other main and interaction effects were not significant (Table 5).

### Effects of SES

#### Personal vs. impersonal dilemmas

With regard to personal and impersonal dilemmas (Table 6), neither main effects of SES nor interaction effects were significant (Table 7).

**Table 6:** **Mean scores (and standard deviations) of personal vs. impersonal dilemmas and moral vs. socio-conventional dilemmas in ANOVA including SES as fixed factor**.

**Group**	**Personal dilemmas**	**Impersonal dilemmas**	**Moral dilemmas**	**Socio-conventional dilemmas**
Medium-low	1.62 (1.55)	2.55 (1.74)	1.37 (2.57)	2.65 (3.20)
Boys	2.00 (1.71)	3.00 (1.78)	2.04 (3.29)	3.35 (3.75)
Girls	1.16 (1.21)	2.00 (1.56)	0.69 (1.29)	1.96 (2.42)
Medium	1.78 (1.49)	2.54 (1.79)	1.95 (3.72)	4.50 (5.21)
Boys	1.92 (1.42)	2.76 (1.79)	2.37 (3.91)	4.66 (5.18)
Girls	1.60 (1.56)	2.26 (1.77)	1.42 (3.43)	4.29 (5.30)
Medium-high	1.92 (1.49)	2.17 (1.65)	1.37 (2.25)	5.02 (4.95)
Boys	2.25 (1.38)	2.39 (1.69)	2.00 (2.72)	5.76 (5.49)
Girls	1.45 (1.54)	1.85 (1.60)	0.55 (0.96)	4.04 (4.06)
Total	1.78 (1.49)	2.45 (1.75)	1.70 (3.22)	4.24 (4.86)
Boys	2.02 (1.46)	2.72 (1.76)	2.22 (3.53)	4.67 (5.04)
Girls	1.48 (1.49)	2.12 (1.68)	1.06 (2.67)	3.70 (4.59)

**Table 7:** **Summary of ANOVA for personal vs. impersonal and moral vs. socio-conventional dilemmas, including SES as fixed factor**.

	**Personal vs. impersonal dilemmas**			**Moral vs. socio-conventional dilemmas**		
	**df**	**F**	**η^2^**	**Df**	**F**	**η^2^**
Kind of dilemmas	1, 203	32.47^***^	0.138	1, 217	102.51^***^	0.321
SES	1, 203	0.21	0.002	1, 217	1.73	0.020
Gender	1, 203	9.00^**^	0.042	1, 217	4.71^*^	0.021
Kind of dilemmas × SES	1, 203	2.50^†^	0.024	1, 217	6.01^**^	0.052
Kind of dilemmas × gender	1, 203	0.01	<0.001	1, 217	0.03	0.001
SES × gender	1, 203	0.53	0.005	1, 217	0.34	0.003
Kind of dilemmas × SES × gender	1, 203	0.38	0.004	1, 217	0.34	0.003

Note:

* *p* < 0.05,

** *p* < 0.01,

*** *p* < 0.001,

† *p* < 0.10.

#### Moral vs. socio-conventional dilemmas

When SES level was introduced into the model as fixed factor (Table 6), the interaction effect of the Kind of dilemma by SES was significant (Table 7). When we computed follow-up ANOVAs separately for mean scores of moral and socio-conventional dilemmas, pupils of different SES levels did not significantly differ in scores of moral dilemmas, whereas, for the socio-conventional dilemmas children from families with middle-low SES had significantly [*F*_(2, 217)_ = 3.12, *p* < 0.05, η^2^ = 0.030] lower scores than peers of middle and middle-high SES. Furthermore, participants with middle-low SES were the ones with the smallest difference between scores for the two types of dilemmas. The difference between scores of moral and socio-conventional dilemmas was significant (*p* < 0.001) in all three SES-level groups, with lower scores for moral dilemmas than for socio-conventional dilemmas. Other main and interaction effects were not significant (Table 7).

## Discussion and conclusions

First of all it is worth noting that the sets of moral dilemmas we devised appeared to be valid overall since the patterns of responses which we recorded were consistent with the underlying theoretical grounds and the literature. As far as personal vs. impersonal dilemmas were concerned, the reluctance to evaluate as acceptable helping an individual by damaging another one was higher if there was a direct contact between the agent and the victim, as compared with situations in which the agent and the victim did not interact directly. As regards the moral type of dilemma compared with the socio-conventional one, the transgression of moral rules was evaluated as less acceptable more often than for socio-conventional rules.

Boys obtained higher scores in the dilemmas than girls, suggesting that girls accepted breaking rules and harming a victim to a lower degree than boys. Literature did not systematically support the notion that boys and girls differ in applying moral criteria to situations or using moral reasoning [for a review, see Killen and Smetana (2006)]. The trends we found in favor of a greater acceptance of rule transgressions and harming actions by boys than girls, however, agrees with the literature on gender differences related to social behaviors, showing that in childhood and adolescence boys are usually more openly aggressive than girls and more inclined to accept aggressive actions (e.g., Rose and Rudolph, 2006) and to self-justify their own violations of moral values (e.g., Hymel et al., 2010).

Moving to the main topic of the paper, in accordance with our hypotheses socio-economic, cultural, and socio-geographic/economic area factors affected the two sets of dilemmas differently. Specifically, all the investigated variables failed overall to affect moral judgments in the personal vs. impersonal dilemmas. Responses in moral and socio-conventional dilemmas, however, were affected by these variables. In particular, children and adolescents in rural areas perceived rules as less breakable than urban peers did and the difference between youngsters living in these two areas was higher for socio-conventional rules than moral rules. Immigrant status significantly interacted with the kind of dilemmas supporting the distinction between moral and socio-conventional tasks: whereas, the native children considered moral rules less breakable than immigrants, the opposite was true for the socio-conventional rules. Such a distinction was stressed also by the effects of SES: whereas, such variables failed to affect moral dilemmas, it appeared that the transgression of socio-conventional rules was judged as acceptable more often by students belonging to families with a middle-high SES status than by students belonging to middle-low SES families.

Overall, the data clearly showed that the investigated socio-economic and cultural differences affected moral evaluations of the transgressions for moral and socio-conventional dilemmas.

### Effects of socio-geographic/economic area, cultural, and socio-economic dimensions on moral reasoning

When we considered our findings in detail, clear evidence emerged that contextual factors can affect at least some kinds of moral reasoning, thus stressing the need to include these dimensions in the investigation of morality even in neuroscience. We found that children and adolescents living in rural areas considered both moral and socio-conventional norms as breakable to a lesser extent than peers living in an urban area. This finding is in line with literature providing evidence that youngsters living in rural areas tend to justify their moral decisions mainly in the norm-following (Nisan and Kohlberg, 1982). This result may express the higher level of social control characterizing small communities like those in the rural area that we investigated. In small towns and villages the compulsoriness of norms can be felt to a higher degree because all the community members contribute to guarantee the respect of the rules (Haidt et al., 2003).

A more complex pattern of results emerged with reference to the immigrant status of participants, as native youngsters considered moral rules breakable to a lesser degree than immigrants, but immigrants accepted the transgressions of socio-conventional rules to a lesser degree than native peers. This finding extends our knowledge of the psychological profiles of immigrant young people (Arredondo-Dowd, 1981). A plausible explanation is that people who have been living since the birth in a country realize, thanks to the knowledge they have acquired of the local or national “history” of some norms, that socio-conventional rules are set and then modified or removed as a consequence of negotiation, and thus they are more aware than immigrants, who do not share the same kind of knowledge, of the non-absolute nature of such rules. An alternative explanation makes reference to the association between social experience and perceived control (Lachman and Weaver, 1998). Since, the environment where immigrants live may be less familiar to them than to native peers, they can perceive a reduced sense of control over such an environment, and this might lead them to perceive it (including the social norms which are part of it) as less modifiable (and thus the social norms as less breakable). However, both explanations are valid for immigrants that have not been living in the host country since a very long time, and that cannot have acquired the same experience of the local culture as the native people.

It worth noting, however, that in general immigrants differentiated less between moral and socio-conventional norms. We have to remember that in our analyses children from different countries of origin and cultural backgrounds were included in the immigrant group (the small numbers of participants belonging to each separate country sub-group prevented us from testing for possible differences among cultures). Thus, we cannot exclude the possibility that the difference in evaluating norms we found is owed not to cultural specificities but to an in-group/out-group effect. That is, it is possible that immigrants feel to a greater extent that all the rules of the host society in which they live need to be respected to the same degree, independently of distinctions between norms.

As far as SES is concerned, children of families of middle-low SES judged socio-conventional rule transgressions as less acceptable than peers whose families had middle and middle-high SES statuses. This outcome is in accordance with findings reported by Haidt et al. (1993) showing that low-SES people considered disrespectful and disobedient actions that were not harmful to others as moral violations. It seems that children and adults of lower levels of SES are less able to perceive the conventionality of rules regulating social behaviors which do not imply injury or personal damage. This may express some differences in the socio-cultural levels of families and of the contexts in which people live and grow up, namely, differences which are tightly bound up with variations in socio-economic conditions. Since, the environment where low-SES children and adolescents live exposes them to less rich sets of experiences, and thus they have fewer opportunities to differentiate among them (Evans, 2004), we have reason to maintain that such reduced differentiation might also apply to behavioral rules. A further explanation, which can be also applied to the difference between native and immigrant persons, refers to possible discrimination effects associated to the status: Children and adolescents belonging to low SES families, as well as immigrants, may be more rigid in conceiving rules because they know or believe that the consequences from infringing them might be heavier—for instance, in terms of social exclusion—for them than the consequences for an individual of a high SES family (or a native person) breaking the same rule (Phinney et al., 1998; Liebkind and Jasinskaja-Lahti, 2000; Calavita, 2007; Brown, 2011).

### Neuroscientific and psychological research on moral reasoning: bridging the theories and the methods

This study is among the first to investigate sensitivity to the distinction between personal vs. impersonal dilemmas among children and adolescents. In accordance with findings provided in the literature on adults, even children and adolescents (as overall sample and separate school-level groups) evaluated actions which harmed an innocent victim for a prosocial purpose as less acceptable when the agent directly interacted with the harmed person than when the contact between the agent and the victim was indirect. This outcome supports the notion that children and adolescents are sensitive, as well as adults, to personal and impersonal distinction. Similarly, Pellizzoni et al. (2010) found that, when administered the simple versions of the switch and the footbridge dilemmas, even the majority of children aged 3–5 years judged the sacrifice of an innocent victim as acceptable when there was no physical contact with the victim and this action was intended to produce the greatest good for the greatest number of people. Along with this result for children of such a young age, our findings on the evaluation of dilemmas by children from primary to high school indicate that the distinction between personal and impersonal persists across different ages.

Similarly to what happened with regard to the personal and the impersonal dilemmas, the distinction between moral and socio-conventional dilemmas was consistent in the overall sample and across the school-level groups: Transgressions of moral rules were evaluated as less acceptable than violations of socio-conventional rules whose purpose was not to preserve others but to guarantee the order of social organization. The similarity of findings on the distinction among personal and impersonal and the distinction among moral and socio-conventional is consistent with the hypothesis of an overlap between the organization of morality as conceptualized in neuroethical studies such as that by Greene et al. (2001) and as conceived in the moral domain theory (Nichols, 2002, 2004). We can speculate that emotions can provide the links between the two theoretical perspectives. Both personal dilemmas and moral dilemmas are likely to activate to a greater extent empathic feelings towards the person who suffers as a result of the agent's action. In situations such as those described in impersonal dilemmas the physical distance between the agent and the victim, which ensues from the indirect contact, does not elicit emotions and empathic responses at the same rate as situations of personal dilemmas. Even more than impersonal dilemmas, the actions represented in socio-conventional dilemmas are likely not to generate empathic emotions, because they are behaviors infringing rules which forbid violations of social conventions without harmful consequences. Therefore, emotions and empathy may underlie differences in moral reasoning and the higher rate of empathy elicited by moral rule transgressions and actions that directly hurt another person may explain the reasons for which at any age (e.g., Smetana and Braeges, 1990; Pellizzoni et al., 2010) these situations are judged to be more serious than socio-conventional rule violations and indirect harmful behaviors, respectively.

The findings about the effects of socio-economic factors on moral evaluations help to complete this picture. Differences in socio-economic and cultural conditions have been found not to affect evaluations of the seriousness of the actions described in personal dilemmas as compared with impersonal dilemmas. On the contrary, socio-economic factors mainly influenced the judgments on infringements of socio-conventional rules, which were considered as more acceptable than moral rule violations. In general, breaking moral rules was scarcely accepted. This pattern of findings supports our hypothesis that personal and impersonal dilemmas may describe two categories of transgressions of the same moral norm which does not allow someone to harm another person. Variations in socio-economic and cultural factors are not influential on differences in moral evaluations of these dilemmas since they express two instances of the same kind of moral violation, which is generally little accepted.

Another novel finding of this study was that the expected higher rates of transgression in the socio-conventional dilemmas in comparison with moral ones emerged in children and adolescents through scenarios that were shorter than those used in traditional research and in which the situation was sketched in a few sentences. These features of the dilemmas we used allow investigators to apply them in research projects realized by means of the fMRI technique, thus making possible studies of moral reasoning as conceptualized by the moral domain theory in the neuroscience framework. This is another possible link between neuroscientific and psychological research on morality. It is worth noting that, even though the structure of Greene et al. (2001) dilemmas and of the dilemmas we used was almost the same, the tasks employed in the present study did not concern, as Greene et al. (2001) dilemmas did, odd situations such as the trolley problem but rather everyday situations. It has been argued that the dilemmas often used in neuroethics investigation concern unusual situations and this is seen as a limitation (Nichols and Mallon, 2006; Klein, 2011). The dilemmas we employed did not share such a limitation, and thus they might be considered for future research.

Besides the links between the neuroethical and psychological perspectives which can be identified at the methodological level, other relationships can be found at the theoretical level. Both perspectives are aimed at discovering the different “signatures” supporting morality (Kelly et al., 2007). In addition, in both paradigms the existence of dual systems has been maintained. Finally, in both perspectives one of the two systems is perceived as closely associated with emotion (Teper et al., 2011). Thus, a series of fundamental similarities (despite obvious differences) can help to construct bridges between the two paradigms. Such bridges can be established thanks to bi-directional relationships. On the one hand, the psychological perspective can propose new conceptual distinctions (such as the distinction between moral vs. socio-conventional rules), show the role played by socio-economic and socio-cultural factors in modulating the corresponding differences, and stress the need for neuroscientific research to detect possible cerebral counterparts. On the other hand, neuroscientific investigations can suggest and support conceptual distinctions on the basis of the evidence of different underlying brain structures and processes. The common goal of discovering the natural grounds of morality might be achieved better thanks to the links between the two perspectives which can be highlighted by applying the same research materials and theoretical frameworks.

### Limitations and future directions

There are some limitations in this study. First, as mentioned before, because of the size of the samples, we could not distinguish in the group of immigrants between children and adolescents from different countries and, consequently, from specific different cultural backgrounds. In addition, the immigrant status as assessed on the basis of the place of birth and on being or not Italian is only an approximated measure of the actual differences in cultures shared by the individuals. These limitations made our interpretation of differences depending on immigrant status speculative to some extent.

Moreover, as regards measures, we have to bear in mind that reliability coefficients for the personal and impersonal dilemmas were low, particularly for the primary school subsample. This feature of the measure may be owed to its short length, but also to the complexity of the assessed construct, for which Greene and colleagues did not provide reliability. Nevertheless, the association we found needs to be cautiously interpreted, because it may be underestimated (Schmitt, 1996). Finally, the collective way in which dilemmas were administered, participants simply being asked to endorse either the “right” or “wrong” response, prevented us from acquiring information about the reasons underlying their responses.

Notwithstanding these limitations, this study was one of the first to provide some bridges between studies on morality in the framework of neuroscience and in the framework of psychological research. In particular, our findings on possible overlaps between the conceptualizations of moral reasoning that were provided by the moral domain theory and the personal vs. impersonal distinction suggest the value of continuing to explore this link between theories. Future studies might investigate the neural counterpart of the distinction between different moral domains, that is, the domains of moral and socio-conventional rules. Future research might also better analyze the possible involvement of emotions in differently evaluating transgressions of moral and socio-conventional norms, even in association with judging as acceptable the harmful action of an innocent victim in the frame of personal or impersonal situations. Even more remarkably, in this study socio-economic and cultural dimensions have been found to be influential on some kinds of moral reasoning. It agrees with the literature, providing evidence that moral knowledge and reasoning, as well as moral behavior (Jimerson et al., 2010), differ as a function of variations in cultures and socio-economic dimensions (Sachdeva et al., 2012). Until now, however, research concerning the neurobiological basis of morality has not considered the possible impact of cultural and economic variation on moral knowledge and behaviors. Nevertheless, a few studies [e.g., Farah et al., 2004; for a review, see Farah et al. (2007)] have started to show that differences in the socio-economic conditions of life are also associated with different activation of neural structures and even possible morphological differences in some areas (Lane et al., 2009). Can socio-economic and cultural factors also affect the neural counterparts of morality? If the answer to the question is positive, do these contextual factors affect differently the neuronal networks involved in the emotional and cognitive facets of morality? These are open issues for future investigation and suggest interesting routes for future research projects trying to fill the gap between distinct fields of study (Killen and Smetana, 2008).

### Conflict of interest statement

The authors declare that the research was conducted in the absence of any commercial or financial relationships that could be construed as a potential conflict of interest.

## Footnotes

^1^In the Italian cultural and linguistic context it is considered discriminatory to ask whether a person is an immigrant, whereas asking a person if she/he is “foreign” is considered neutral. This cultural specificity explains why in surveys realized in Italian contexts adults and children are usually asked to report whether they are “foreign,” in comparison with “Italian,” and not “immigrant.”

^2^The ANOVAs which were performed separately for the three school-level groups confirmed that the school-level did not moderate the associations between the investigated variables, thus, these analyses were not further considered.
